# STEADI for Falls Prevention in Outpatient Rehabilitation: An Outcomes Evaluation Using the RE-AIM Framework

**DOI:** 10.1093/geroni/igaf122.294

**Published:** 2025-12-31

**Authors:** Jennifer Vincenzo

**Affiliations:** University of Arkansas for Medical Sciences, Little Rock, Arkansas, United States

## Abstract

We used the RE-AIM (Reach, Effectiveness, Adoption, Implementation, and Maintenance) framework to identify the implementation outcomes of the Stopping Elderly Accidents, Deaths, and Injuries (STEADI) Initiative in 34 outpatient rehabilitation clinics within a health system between 2018–2021. STEADI was used to screen and assess all older adults ≥65 years for falls risk. STEADI Implementation strategies included health system mandates, EHR revisions, email instructions, educational sessions and resources, clinical leads and champions, and chart audits. Using Electronic Health Records (EHRs) and descriptive statistics, we identified the Reach, Adoption, and Maintenance of screenings and physical assessments to identify fall risk among older adults.

**Reach:**

76.4% (50,023) of older adults seen by a rehabilitation provider had a completed screening, and 44.1% screened at risk for falls.

**Adoption:**

Clinic-level adoption varied, with most performing screenings. Profession-level adoption was highest for physical therapists (PTs; 94.2% initiated, 80.6% completed) and lowest for speech-language pathologists (SLPs; 79.8% initiated, 55.9% completed).

**Reach and Adoption of functional outcomes measures (FOM):**

PTs completed a FOM on 59.5% of at-risk patients, occupational therapists on 11.6%, and SLPs on 7.9%.

**Maintenance:**

All measures declined 1%–10% annually between 2018 and 2021. STEADI screening and FOMs were implemented systemwide in 34 outpatient rehabilitation clinics, reaching over 50,000 older adults. Screening adoption rates varied by clinic. PTs had the highest adoption rate. All adoption rates declined over time. Future research should consider an implementation science approach with input from key partners to identify barriers and develop strategies to support all STEADI components in outpatient rehabilitation.

